# Pathological Human Tau Induces Alterations in the Brain Insulin Signaling Cascade

**DOI:** 10.3389/fnins.2022.805046

**Published:** 2022-02-21

**Authors:** Abdeslem El Idrissi, Alejandra del Carmen Alonso

**Affiliations:** ^1^Department of Biology and Center for Developmental Neuroscience, College of Staten Island, The City University of New York, New York, NY, United States; ^2^Biology Program, The Graduate Center, The City University of New York, New York, NY, United States

**Keywords:** Alzheimer’s disease, hyperphosphorylated tau, tau transgenic mouse model, seizures, insulin resistance, brain waves, glucose tolerance test

## Abstract

The process of neurodegeneration in Alzheimer’s disease has been associated with a disruption of insulin signaling cascade in neurons, and to insulin resistance. T2DM correlates with Alzheimer’s disease, but mechanisms of interaction are unknown. We have developed a mouse model of tau induced neurodegeneration expressing pseudo-phosphorylated tau [Pathological Human Tau (PH-Tau)] in neurons. This model (PH-Tau-Tg) recapitulated cognitive decline and neurodegeneration observed in AD. In this study we examined if expression of PH-Tau could affect neuronal excitability and insulin receptor signaling. Neuronal excitability was investigated using intracerebral recordings of extracellular field potentials from prefrontal cortex after insulin and kainic acid (KA) injection. Analysis of baseline recordings indicated an increased excitability of PH-Tau-Tg as evidenced by higher spectrum densities (PSDs) of high frequencies brain waves. Injection of insulin (1IU, s.c) led to a decrease of fast ripples PSDs, more pronounced in PH-Tau-Tg mice than controls. Subsequent injection of kainic acid (KA, 5 mg/kg, s.c) led to significant increase in firing rate, amplitude of extracellular field potentials and PSDs of high frequency brain waves in control mice only. To further investigate the role of insulin in PH-Tau-Tg mice, we subjected mice to a glucose tolerance test. We found that PH-Tau-Tg mice were significantly hyperglycemic prior to glucose injection. Interestingly, the PH-Tau-Tg mice showed a moderate increase at 30 min due to the higher baseline, indicating a low sensitivity of insulin receptor in these mice. This is consistent with increased levels of activated insulin receptors in the brain and the inhibitory effect of insulin on ictal activity post KA injection in PH-Tau-Tg mice. We suggest that these mice have reduced insulin sensitivity (hyperglycemia) and as a compensatory mechanism there is overactivation/expression of insulin receptor in the brain rendering neuronal circuits resistant to seizure induction after injection of insulin. These data indicate that insulin signal transduction pathway is altered in PH-Tau-Tg mice, and that injection of exogenous insulin reduces hypersynchronous bursting activity of field potentials recorded from cortical neuronal circuits. We propose that the appearance of abnormal tau might potentiate the toxic environment by interfering with the insulin signaling cascade in the brain of patients with Alzheimer’s disease.

## Highlights

– Transgenic mice expressing pathological human tau (PH-Tau) in the neurons exhibit hyperexcitability of neuronal circuits.
– PH-Tau-tg mice also exhibit features of type 2 diabetes, and are hyperglycemic.
– Insulin signal transduction pathway is altered in PH-Tau-Tg mice and have elevated expression of pS312 insulin receptors in the brain.
– Injection of exogenous insulin reduces hypersynchronous bursting activity from cortical neuronal circuits PH-Tau-Tg mice.
– Abnormal tau might potentiate the toxic environment by interfering with the insulin signaling cascade in the brain.

## Introduction

Tauopathies are a group of neurodegenerative diseases characterized by the accumulation of hyperphosphorylated tau in the presence or absence of other lesions. These include Alzheimer’s disease (AD), Fronto-Temporal Dementia, Chronic Traumatic Encephalopathy, Pick’s Disease, tangle-only dementia to list a few (Lee et al., 2001; Iqbal et al., 2009; McKee et al., 2009; Murray et al., 2015; Alonso et al., 2018). Alzheimer’s disease (AD) is a devastating neurodegenerative disorder affecting roughly 30 million people worldwide. AD is characterized by a cascade of pathological events, including the formation of amyloid plaques (made up of aggregated forms of Aβ), neurofibrillary tangles (composed of aggregated, hyperphosphorylated tau), synapse loss, brain hypometabolism, neuroimflammation, and brain atrophy that is accompanied by severe and progressive cognitive decline. Amyloid plaques, the other hallmark of AD are generated when Aβ peptide aggregate and accumulate in the extracellular space. The buildup of hyperphosphorylated and aggregated tau protein leads to the development of intracellular neurofibrillary tangles. Many factors, genetic and non-genetic, have been identified in the etiology of AD. Amongst them, type 2 diabetes (T2D), a disease of aging, has been shown to increase the risk for AD risk (Sims-Robinson et al., 2010).

Insulin receptors are widely expressed in the brain. Their regional specificity and the complexity of insulin signal transduction makes the effects of insulin on the brain pleiotropic (Unger et al., 1991). However, glucose utilization in the brain is insulin independent.

Insulin is primarily a metabolic hormone functioning on muscle, fat and liver *via* activation of insulin receptor (IR). Once insulin is secreted, it crosses the blood-brain barrier by a transporter-mediated saturable mechanism (Banks et al., 1997). Several studies have implicated IR activation in the regulation of excitatory and inhibitory neurotransmission. The expression of IR in the brain is activity-dependent (Plum et al., 2005). IR regulate the expression of potassium ion channel Kv1.3 in the olfactory bulb after intranasal insulin injection to mice (Marks et al., 2009). This led to increase to enhancement of memory in these mice (Marks et al., 2009), suggesting a cognitive enhancing role for insulin through activation of IR and increased expression of Kv1.3 channels. Additionally, insulin prevents cells death of hippocampal neurons deprived of glucose *in vitro* (Mielke et al., 2006). Insulin signaling in the brain has been shown to be important for both metabolic homeostasis and higher brain functions such as cognition. Insulin reduces brain excitability by lowering the threshold for extrasynaptic GABA_A_ receptors activation, increasing therefore the GABA-mediated tonic inhibitory conductance (Jin et al., 2011). Impaired insulin signaling increases risk of Alzheimer’s disease (Ronnemaa et al., 2008), cognitive disabilities in diabetes mellitus (Seaquist, 2010) whereas intranasal administration of insulin improves hippocampal-dependent memory function (Benedict et al., 2007).

Several studies provide significant insights and experimental evidence on the mechanistic link between AD and T2D and show the reciprocal actions between these two diseases, suggesting a shared common cellular and molecular mechanisms (Han and Li, 2010). In AD, it has been reported brain insulin resistance as an early sign of cognitive impairment and increase levels of Ser phosphorylated IRS-1 has been shown to precede cognitive decline in AD (Talbot et al., 2012). Insulin signaling alterations have been reported in the different mouse models of AD, like the triple transgenic mouse model or the APP/PS1 transgenic model or the one induced by the intracerebroventricular (icv) injection of amyloid-β oligomers (reviewed in Lyra e Silva et al., 2019). Changes in brain insulin resistance had been attributed to Abeta amyloid accumulation. But if the presence of hyperphosphorylated tau is related to insulin resistance is less explored. Activation of the insulin pathway has been related to increase in tau hyperphosphorylation, and it is currently viewed that tau hyperphosphorylation might be a consequence of an altered insulin signaling transduction pathway. Some studies are reporting metabolic alterations in the tau transgenic mice, like in a knocked in tau model that is hyperglycemic at 8 months of age and higher levels of phosphorylated tau were detected (Hull et al., 2020). Increase brain response to insulin was reported in a transgenic mouse model expressing a double FTDP-17 tau mutant (Leboucher et al., 2019). Furthermore, metabolic changes such as lower levels of leptin and insulin and resistance to high-fat diet were reported. Recently, the same group reported increased seizures susceptibility and excitability in the same tau transgenic model (Gomez-Murciaa et al., 2020). These are very intriguing observations, however, it should be considered that the level of transgenic tau expression in these animals is four to five times higher than tau endogenous levels at 3 months old animals and five to six-fold higher at 12 months of age (Schindowski et al., 2006).

We have demonstrated that a PH-Tau form (PH-Tau, R406Wtau pseudo-phosphorylated at Ser 199, Thr212, Thr231, and Ser262) mimics AD abnormal tau (Alonso et al., 2010), impairs learning and memory in Drosophila (Beharry et al., 2013) and in a transgenic mouse model in which it induces neuronal death and astrocytes activation (Di et al., 2016). In our transgenic mouse model PH Tau is expressed only in the neurons, this is a bigenic mouse model where the expression is controlled by the expression of a trans-activator, tTA, controlled by the CaCamII promotor, inducing the expression in neurons. In this model we can prevent the expression of PH Tau with doxycycline, but for this report, all the mice were raised with food without the antibiotic. Under these conditions, PH Tau is expressed in all developmental stages but the levels of expression do not exceed 14% of the endogenous tau expression with no decrease on the native tau expression. Mice expressing PH Tau showed cognitive impairment, as well as neuronal death in the hippocampus and tau aggregation at 12 months of age. In the present report we set up to investigate the insulin signaling pathway in our mouse model of neurodegeneration in adult mice but at an earlier age than when we observed the structural changes in the brain. Therefore we chose 8 months of age to test the insulin signaling pathway, and we show that low level of expression of PH-Tau is enough to induce brain insulin resistance.

## Materials and Methods

### Animals

A total of 24 males mice were used in this study. The control mice were 6-month-old C57BJ6 × 129 males. The PH-Tau-tg mice 8-months old mice were generated as described (Supplementary Material, Di et al., 2016). All mice were housed in groups of three-to-four in a pathogen-free room maintained on a 12 h light/dark cycle and given food and water. All procedures were approved by the Institutional Animal Care and Use Committee of the College of Staten Island/CUNY and were in conformity with National Institutes of Health Guidelines.

### Drug Administration

Kainic acid and Insulin were dissolved in isotonic saline and injected at 5 mg, kg^–1^ and 1IU, kg^–1^, respectively. Animals received the indicated drugs through a stationary butterfly needle inserted subcutaneously before the start of the recordings and attached to a 20 cm catheter to avoid any electrostatic artifacts during the injections. After each injection, the catheter was flushed with a small volume of saline so the whole dose of the drug could be delivered.

### Intracerebral Recordings of Local Field Potentials

Mice were anesthetized with ketamine/xylazine mix (90/10 mg, kg^–1^ i.p.), scalps were shaved, then fixed on a stereotaxic apparatus. Right side craniotomies were made at AP 2.5 mm from bregma, L 0.5 mm (medial prefrontal cortex). Extracellular recordings were obtained with tungsten electrodes with impedances of 1–2 MΩ. Electrodes were placed in infragranular layers (0.5 lateral and 1.0–1.2 mm deep in prefrontal cortex). Local field potential (LFP) from the pre-frontal cortex were recorded. LabChart-8 (ADInstruments, Colorado Springs, CO, United States) was used for LFP recording this includes both frequency domain and time domain features that have been extracted. The low-frequency oscillations (LFO): delta 0.4–4 Hz, theta 5–7 Hz, alpha 7–12 Hz, beta 13–25 Hz, gamma 26–80 Hz. High frequency oscillations (HFO): slow ripples 125–250 Hz and fast ripples 250–500 Hz. All recordings were passed through a preamplifier connected to the electrode and amplified using model 1700 differential AC Amplifier (ADInstruments) and digitized at 10 kHz.

### Immunohistochemistry

Cryosections were made and placed onto gelatin-subbed slides. Non-specific binding sites were blocked using 4% bovine serum albumin (BSA), 2% normal goat serum (NGS), and 0.05% Triton X-100 in 0.01 M phosphate-buffered saline (pH 7.2). Following the blocking step, the slides were rinsed in an antibody dilution cocktail (ABD) consisting of 2% BSA and 1% NGS in 0.01 M PBS. Primary antibodies (Life Sciences) employed were directed against the phosphorylated (activated) insulin receptor phosphor IR substrate 1 (p-Ser312-IRS1; Rabbit polyclonal) and Glucose transporter (Glut4; Mouse monoclonal) diluted 1:500 in ABD. The primary antibodies were incubated overnight at 4°C and then unbound antibodies rinsed with ABD. Secondary antibodies were all raised in goat and directed against appropriate primary antibody type. The anti-mouse IgG was conjugated to Cy5 and anti-rabbit was conjugated to Cy3 (1:500; Invitrogen/Molecular probes). Sections were rinsed in PBS and coverslipped with VectaShield mounting medium with DAPI (Vector Labs, Burlingame, CA, United States). Coverslips were sealed using clear nail polish (Electron Microscopy Sciences, Ft. Washington, PA, United States). Slides were stored in an opaque slide box at 4°C temperature until imaging. Images were obtained by confocal microscopy (Leica SP2 AOBS). Z stack images were acquired using a Plan-Apochromat 63X/1.4 oil objective. Stacks were collected at a 0.5 μm slice interval, stepping through the entire section. Frame size was set to 1024 × 1024 pixels. All image acquisition parameters including gain and offset were identical for all comparisons. Z-stacks were opened in Imaris in their native format. And automatically reconstructed into a multi-channel 3D model during input into Imaris, requiring no further image pre-processing. Background subtraction was used to separate immunoreactivity from the background signal. The auto-threshold value was utilized during background subtraction, without user adjustments. The size and shape of the generated surface were a direct map of the intensity distribution of p-Ser312-IRS1 and Glut4 immunolabeling and DAPI labeling within the sections as detected by Imaris. To quantify immunoreactivity, the Spots creation tool was used. Estimated XY diameter for Spot detection was 0.5 μm. To determine relative changes in protein expression, all quantification parameters initially set up for control z stacks were applied to the z stacks obtained from PH-Tau-Tg brain images. Changes in expression were also confirmed statistically using the Imaris ×64 software (Bitplane). Statistical significance was set at (*p* < 0.05).

### Intraperitoneal Glucose Tolerance Test

Mice from both groups were fasted overnight (12 h) and then injected intraperitoneally with 0.02 ml/g of body weight D-glucose (7.5% stock solution in saline). Blood samples were taken by tail venesection at 0 min (just before glucose injection) and at 30-, 60-, and 120-min intervals after the glucose load. Glucose was measured with Ascensia Breeze portable glucose meter (Bayer, Leverkusen, Germany). Mice were given only water during the test.

### Statistical Analysis

The mean power spectral density was compared by generalized linear mixed model with log normal distribution. The fixed effects were modeled as treatment, treatment*genotype for each frequency band. The model was adjusted for inter electrode variability nested on animals using variance components from random effects. Tukey–Kramer was used as multiple comparisons test. Electrophysiological results are shown as the mean of each parameter, significance values were determined by one-way repeated measures ANOVA and a *post hoc* Dunnett test with *p* < 0.05. Peak amplitude of the response was calculated using LabChart software (ADInstruments, Colorado Springs, CO, United States) and data was analyzed using SPSS 8.0 software.

## Results

### Insulin Reduced the Amplitude and Frequency of KA-Induced Epileptiform Discharge in PH-Tau-Tg Mice

**FIGURE 1:**
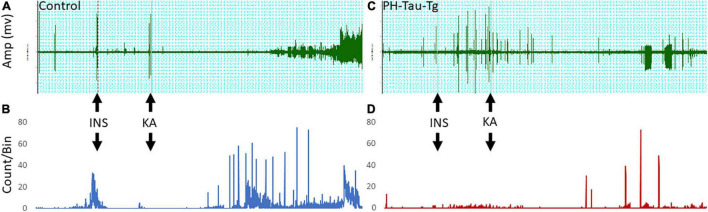
Representative intracerebral recording from the prefrontal cortex of a control mouse **(A)** and PH-Tau-Tg mouse **(C)**. Recordings were performed for 15 min (baseline) followed by an i.p. injection of insulin (1IU) at 10 min later and KA (5 mg/kg) at t25. Onset of high frequency discharge and ictal activity occurred about 20 to 30 min post KA injection. In controls, once seizures occurred there was no recovery and the amplitude of the population spikes almost doubled and remained so for the duration of the recordings. In PH-Tau-Tg mice the latency was the same as controls, however, the amplitude of the population spikes was significantly lower and ictal activity was intermittent and interrupted with interictal events **(C)**. Spike histograms analysis of firing rates with a detection threshold of 0.1 mv and a Bin size of 1 s showed a significant increase after KA injection in controls **(B)** and drastic decrease post KA-injection in PH-Tau-Tg mice **(C)** (*n* = 3 of each group) Shown here are representative intracerebral recordings from individual mice.

In this study, we examined electrophysiologically the effects of insulin on neuronal excitability and seizure susceptibility in control and PH-Tau-Tg mice using the neurotoxin KA. KA activate a subtype of glutamate receptors (KA receptors), which are densely present in hippocampal and cortical interneurons and other principal neurons (Monaghan and Cotman, 1982; Wisden and Seeburg, 1993). KA receptors are channels that regulate Na^+^, K^+^ and Ca^2+^ conductance, responsible for fast synaptic transmission through generation of excitatory postsynaptic currents (EPSCs; Carta et al., 2014; Cossart et al., 2002; Ruiz et al., 2005). Activation of these receptors by KA, which trigger a cascade of events resulting in seizures, is the basis for the temporal lobe epilepsy model (El Idrissi et al., 2003). At a dose of 5 mg.kg^–1^, KA first produced seizures with no motor expression and recorded as regular EEG spiking (Figure 1). Occasionally, these seizures ended with rapid tail shakes and could be followed by a recurrent seizure with high-frequency spikes and no behavioral concomitants. Here, we examined the effects of insulin receptor (IR) activation on neuronal excitability by quantifying the amplitude and frequencies of epileptiform discharges following KA injection. We injected insulin and subsequently injected low doses (5 mg/kg) of KA, a depolarizing agent with preferential binding to limbic structures. Synaptic activity triggers membranes currents that pass through the extracellular space and is measured by electrodes placed outside the neurons as local field potentials (Figures 1A,C upper traces). In control mice, intracerebral recording revealed epileptiform discharges about 20 min post KA injection (Figure 1A). Spike histogram analysis of recorded field potentials show a significant increase [*t*(4,712) = 19.28, *p* < 0.001] in the firing rate post KA injection, varying from 20 to 70/s (Figure 1B). In PH-Tau-Tg mice, however, addition of insulin led to an increase in the amplitude and frequency of population spikes and subsequent addition of KA in the presence of insulin resulted in a significant increase (*p* < 0.01) in the latency to the onset of epileptiform discharge, a significant decrease (*p* < 0.01) in the frequency of ictal events and an overall decrease in the amplitude and frequency of population spikes (Figure 1C). Spike histogram analysis of recorded field potentials shows a significant reduction in the firing rate in the presence of KA in the PH-Tau-Tg mice compared to controls [*t*(4,712) = 19.28, *p* < 0.001] (Figure 1D).

### Insulin Suppresses KA-Induced Epileptiform Discharge and High Frequency Oscillations of Brain Waves in PH-Tau-Tg Mice

**FIGURE 2:**
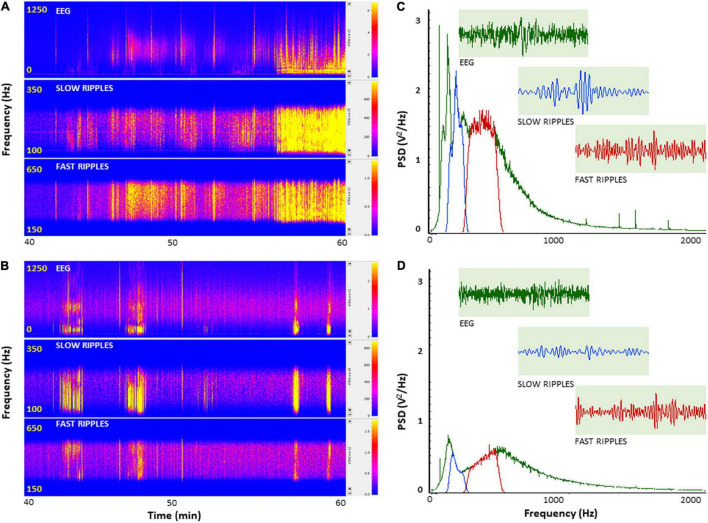
Insulin selectively depresses cortical ripples in PH-Tau-Tg mice brains. PSDs analysis of field potential recordings from prefrontal cortex after KA injection. FFT of population spikes and filtered high frequency oscillations (HFO): slow ripples 125–250 Hz and fast ripples 250–500 Hz are shown in **(C,D)** with representative traces (inserts). The power density of field potentials and filtered frequencies were converted into heat maps **(A,B)**, Controls and PH-Tau-Tg mice, respectively). **(A,B)** Are representative heat maps of intracerebral recording, slow ripples and fast ripples from a control mouse **(A)** and PH-Tau-Tg mouse **(B)**. **(C,D)** Are FFT of PSDs obtained from pooled data of controls (*n* = 3) and PH-Tg mice (*n* = 3) respectively.

To further investigate how PH-Tau affects the function of neuronal circuits and their firing properties, we examined neuronal excitability by measuring cortical field potentials from mice treated with KA in the presence of insulin. Quantitative analysis of the power spectral density (PSD) obtained from the recorded field potential traces demonstrate a significant increase in neuronal firing from controls compared to PH-Tau-Tg mice *F*(1) = 185.59, *p* < 0.001 (Figure 2C). Segment analysis reveals that most of the increase in firing frequencies occurred in the presence of KA (Figures 1A,C), Therefore, we focused our quantitative analysis post KA injection and starting at 40 min into the recording. Peak increase in PSD amplitude could be seen around 100–600 Hz (Figure 2C). This range of high frequency oscillations (HFO) encompasses slow ripples 125–250 Hz and fast ripples 250–500 Hz as shown in Figures 2C,D. Therefore, field potential traces were filtered between 125 and 500 Hz and amplified. Ripple oscillations are HFOs ranging from 125 to 500 Hz, and they have been regarded as a potential marker of epileptogenicity (Bragin et al., 1999, 2000, 2004; Traub et al., 2002; Grenier et al., 2003; Dzhala and Staley, 2004; Jacobs et al., 2008; Zijlmans et al., 2009; Brázdil et al., 2010; Haegelen et al., 2013; Wang et al., 2013; Miao et al., 2014). Segment analysis revealed a significant increase in the PSD of slow ripples [*t*(328) = 6.79, *p* < 0.001] and fast ripples [*t*(656) = 11.88, *p* < 0.001] (Figures 2C,D) with the addition of KA, consistent with its epileptogenic effect. These increases were significantly suppressed by insulin in the PH-Tau-Tg mice *F*(1) = 185.59, *p* < 0.001 (Figure 2D). Segment analysis reveals that most of the increase in PSD of HFO was in the presence of KA. Fourier transform (FFT) analysis of raw data showed a significant increase in peak activity of both HFOs consistent with increased neuronal excitability (Figures 2C,D). PSD analysis of population spikes show a significant decrease of peak amplitude from PH-Tau-Tg recordings in the presence of insulin compared to controls *F*(1) = 185.59, *p* < 0.001 (Figures 2C,D). Insulin significantly suppressed KA-induced increase in peak amplitude of PSDs and in both the slow [*F*(1) = 81.48, *p* < 0.001] and fast [*F*(1) = 9.95, *p* < 0.001] ripple oscillations suggesting a role in elevating seizure threshold in the PH-Tau-Tg brains. These data clearly indicate that field potentials recorded from cortical neuronal circuits in control mice reflect hypersynchronous bursting activity consistent with hyperexcitability. Insulin significantly suppressed hypersynchronous firing in the brain of PH-tau-tg mice.

### Insulin Suppresses Cortical Ripples in PH-Tau-Tg Mice

**FIGURE 3:**
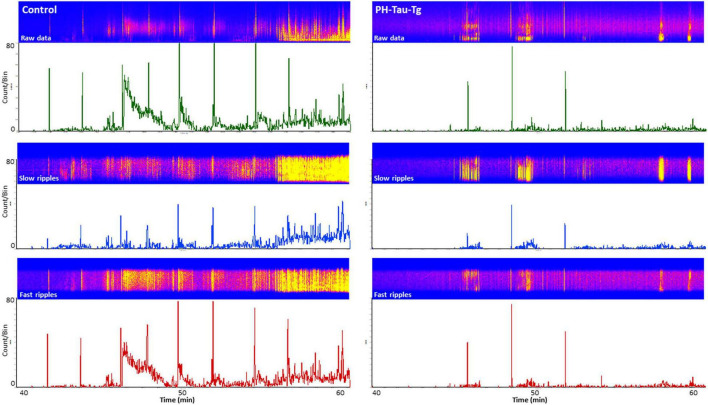
Selective enhancement of cortical ripples after KA injections. Firing rate analysis with a detection threshold of 100 μv shows a significant increase after KA injection in controls **(left)** and a drastic decrease in PH-Tau-Tg mice **(right)**. Spike histograms of filtered frequencies show a significant increase in both slow ripples [*F*(1) = 81.48, *p* < 0.001] (125–250 Hz) and fast ripples *F*(1) = 9.95, *p* < 0.001 (250–500 Hz) post KA injection in controls in presence of insulin (left panel). Insulin injection led to a significant suppression of both HFOs post KA injection. Detection threshold for slow ripples and fast ripples were 0.02 and 0.04 mv, respectively. Bin size was set to 1 s. The power density of field potentials and filtered frequencies were converted into heat maps and shown on top of the respective spike histogram (left, Controls and right, PH-Tau-Tg mice). Heat maps are representatives of a control mouse and PH-Tau-Tg mouse. Spike histograms are obtained from pooled data of controls (*n* = 3) and PH-Tg mice (*n* = 3) respectively.

To further investigate the effects of insulin on neuronal excitability and seizure susceptibility, we analyzed the rate and amplitude of population spikes post KA injection, specifically during ictal activity (last 20 min of the recordings). Firing rate analysis with a detection threshold of 0.1 mv showed a significant increase [*t*(4,712) = 19.28, *p* < 0.001] in the firing frequency after KA injection in controls (as high as 80 population spikes/second, upper trace, Figure 3). On the other hand, recordings from PH-Tau-Tg mice had a significantly lower rate (upper right trace, Figure 3). Spike histogram analysis of filtered frequencies showed a significant reduction in the firing rate of both slow ripples [*t*(328) = 6.79, *p* < 0.001] (125–250 Hz) and fast ripples [*t*(656) = 11.88, *p* < 0.001] (250–500 Hz) post KA injection in the PH-Tau-Tg mice injected with insulin (Figure 3).

### Insulin Reduces Seizures Severity and Propensity in PH-Tau-Tg Mice

**FIGURE 4:**
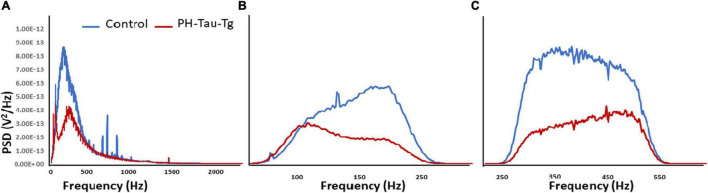
Insulin reduces KA-induced increased in PSDs in PH-Tau-Tg mice. Field potential recordings from prefrontal cortex were filtered at the indicated frequencies and amplified and the respective power spectrum densities are shown above. **(A)** Represents the power spectrum density of the recording of field potentials obtained from controls (*n* = 3) and PH-Tau-Tg mice (*n* = 3) in response to addition of KA. **(B,C)** Represent the power spectrum density of slow ripples (125–250 Hz) and fast ripples (250–500 Hz) obtained from recordings after addition of KA. Insulin significantly reduced the amplitude PSDs for HFOs in the PH-Tau-Tg mice. Data represent means of pooled recordings from controls (*n* = 3) and PH-Tg mice (*n* = 3) respectively.

Quantitative analysis of the power spectral density of the filtered frequencies obtained from the recorded field potential demonstrate a peak frequency distribution in neuronal firing after addition of KA around 100–500 Hz. Power spectrum calculates the area under the signal plot using the discrete Fourier Transform, the power spectrum density assigns units of power to each unit of frequency (Figure 4A). This frequency range correspond to HFOs encompassing slow ripples and fast ripples (slow ripples 125–250 Hz and fast ripples 250–500 Hz). Slow and fast ripple waves are commonly used as a biomarker of epileptogenic brain. Figure 4 shows PSDs obtained from segment analysis of field potentials, slow ripples and fast ripples. As shown in Figure 4, addition of KA led to a selective increase in the power spectrum of HFOs corresponding to the ripples (slow and fast) with a significant difference in peak amplitude between controls and PH-Tau-Tg mice [*t*(8,190) = 10.80, *p* < 0.001]. Consistently, PDSs analysis of filtered HFOs (slow ripples 125–250 Hz and fast ripples 250–500 Hz) showed a significant increase in the amplitude of their power spectrum densities (Figures 4B,C, respectively) with the addition of KA [*F*(1) = 185.59, *p* < 0.001]. Injection of insulin prior to KA injection led to a significant decrease in PSD peak amplitude of both field potentials and HFOs [Slow ripples *t*(328) = 6.79, *p* < 0.001; fast ripples *t*(656) = 11.88, *p* < 0.001]. These data clearly indicate that insulin reduces hypersynchronous bursting activity and hyperexcitability of neuronal circuits of the PH-Tau-Tg mice. We suggest that the higher threshold for KA-induced seizures in PH-Tau-Tg-injected mice is due to an increase in GABA receptor function in the brain which increases the inhibitory drive rendering neural circuits seizure-resistant and might be mediated by direct modulation the GABA_A_ receptors *in vivo*. This is supported by the finding that insulin reduces brain excitability by lowering the threshold for extrasynaptic GABA_A_ receptors activation, increasing therefore the GABA-mediated tonic inhibitory conductance (Jin et al., 2011).

### Altered Insulin Signaling in the PH-Tau-Tg Brains

**FIGURE 5:**
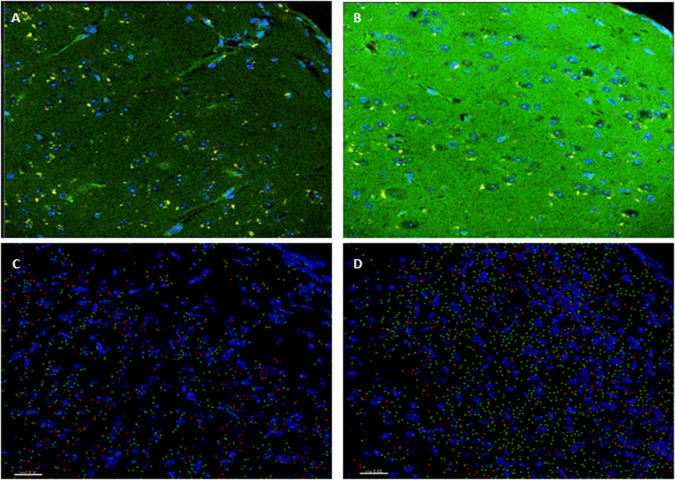
PH-Tau-Tg mice have elevated levels of expression of activated insulin receptor and glucose transporter. **(A)** Control and **(B)** PH-Tau-Tg are representative images of a 30 μm cryosection probed with anti-p(Ser)-IRS1 (green) and anti-Glut 4 (red) showing the pattern and intensity of immunoreactivity in the cortex. DAPI (blue) was included in the mounting medium and used for nuclear localization. **(C)** Control and **(D)** PH-Tau- are Images that depict Imaris reconstructions of the z-stacks obtained with a confocal microscope of the same regions shown in **(A,B)**. Cortex from PH-Tau-Tg mice shows a significant (*p* < 0.05) increase in immunoreactivity for p(Ser)-IRS1 and Glut 4. Scale bar = 20 μm.

Insulin signaling in the brain has been shown to be important for both metabolic homeostasis and higher brain functions such as cognition. Impaired insulin signaling increases risk of AD and cognitive disabilities in diabetes mellitus. To further investigate the involvement of insulin in the modulation of neuronal excitability, we examined the expression patter of the insulin receptor in the cortex. Interestingly, higher level of phosphorylated insulin receptor (p-Ser-IRS1) has been shown to be a consistent change in insulin signaling and a marker of insulin resistance in AD brains (Steen et al., 2005; Moloney et al., 2010; Bomfim et al., 2012; Talbot et al., 2012; Yarchoan et al., 2014). p(Ser)-IRS1 is a substrate for p-JNK, which also has also been found in elevated levels in AD brains (Bomfim et al., 2012; Talbot et al., 2012), suggesting some level of insulin resistance in AD. Consistent with this, we found a significant increase p(Ser)-IRS1 in PH-Tau-Tg mice compared to controls (*p* < 0.05) (Figure 5). Concomitant with the increase in p(Ser)-IRS1 in PH-Tau-Tg cortices, we also found a moderate increase in the expression levels of Glut 4. The expression levels of Gluts 4 has been shown to be dependent on the activation levels of IR (Figure 5).

### PH-Tau-Tg Mice Are Hyperglycemic

**FIGURE 6:**
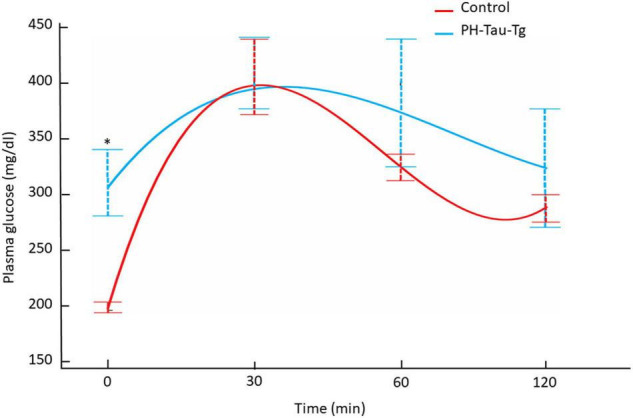
Intraperitoneal glucose tolerance test on overnight fasted control mice (*n* = 7) and PH-Tau-Tg mice (*n* = 9). Values are expressed as means ± SEM obtained from three experiments. *p < 0.05 when compared with control group.

To further investigate the role of insulin in PH-Tau-Tg mice, we subjected the mice to a glucose tolerance test. Mice were fasted for 12 h and injected with a glucose solution (7.5 mg/kg, s.c) then plasma glucose levels were monitored for 30, 60 and 120 min post injection. We found that PH-Tau-Tg mice were significantly hyperglycemic prior to glucose injection (Figure 6; baseline; *p* < 0.05). As expected, peak plasma glucose was observed at 30 min post glucose injection and at 120 min mice became normoglycemic. At 60 and 120 min PH-Tau-Tg mice glucose plasma levels were slightly but not significantly higher than controls, indicating a low sensitivity of insulin receptor in these mice. This is consistent with increased levels of activated insulin receptors in the brain and the inhibitory effect of insulin on ictal activity and epileptiform discharge post KA injection in PH-Tau-Tg mice.

## Discussion

In this study, we used a PH-Tau-Tg mouse to investigate the alteration in insulin signal transduction pathway and the resulting functional significance on neuronal excitability and seizure susceptibility. Insulin has been shown to play a pleiotropic role in the brain. Insulin receptors (IR) and have been identified in several regions of the brain that mediate important physiological effects, such as neuronal development, glucose uptake regulation, feeding behavior, and body weight, as well as cognitive processes, including attention, executive functioning, learning, and memory (Derakhshan and Toth, 2013). IRs are widely distributed throughout the brain (Fernandez and Torres-Alemán, 2012) and their expression is largely localized to neurons (Unger et al., 1989), although IR mRNA is present in glia and endothelial cells (Zhang et al., 2014). Insulin is a neuromodulators on mammalian CNS by affecting the function of certain neurotransmitter receptors therefore affecting electrophysiological properties of neurons and neuronal circuits. Insulin may play an important role in the control of GABA receptor density in the post-synaptic domain (Wang et al., 2003). Insulin also affects intracellular ion concentrations by modulating the activity of certain ion channels. In hypothalamic neurons, insulin activates ATP-dependent K^+^ channels leading to membrane hyperpolarization, mechanistically similar to those in the β cells of the islets (Plum et al., 2005). In addition, insulin has stimulatory effects on Na^+^/K^+^ ATPase, producing an acute rise in intracellular Ca^2+^ concentration that triggers the release of inhibitory neuropeptides under high frequency firing (Jonas et al., 1997). There is also experimental evidence that insulin affects learning and memory through GABA receptors by stimulating the translocation of these receptors to the plasma membrane. This effect is abolished by the action of a PI3K inhibitor. Insulin also increases the functional GABA receptor expression on the post-synaptic and dendritic membranes of the CNS neurons (Ghasemi et al., 2013). Insulin also modulates glutamatergic neurotransmission at the synapses. This hormone induces long term depression by decreasing the amount of AMPA receptors in the post-synaptic membrane. This process is mediated by activation of insulin receptor and downstream activation of PI3-kinase (Huang et al., 2004). Furthermore, insulin has been shown to induce the phosphorylation of the GluR2 subunit in the AMPA receptors of hippocampal neurons leading to their internalization and a decrease in EPSPs amplitude and neuronal excitability (Ahmadian et al., 2004). Therefore, it is well established that insulin affects many aspects of neuronal development, metabolic homeostasis, and higher brain functions such as cognition. Disturbances of these processes through Impaired insulin signaling can lead to many pathological conditions including increased risk of AD and cognitive disabilities in T2D.

To further investigate the role of insulin in the regulation of neuronal excitability we pre-injected mice with insulin and measured the electrophysiological responses to KA. At a dose of 5 mg.kg^–1^, kainate first produced seizures with no motor expression and recorded as regular EEG spiking, varying from 60 to 80/s (Figure 1). These filed potentials are a result of synaptic activity that triggers membrane currents that pass through the extracellular space and are recorded by electrodes placed outside the neurons. Field potential traces were filtered between 125 and 500 Hz and amplified. We focused our analysis on High frequency oscillations (HFO): slow ripples 125–250 Hz and fast ripples 250–500 Hz as shown in Figures 2–4. Ripple oscillations are HFOs ranging from 125 to 500 Hz, and have been regarded as a potential biomarker of epileptogenicity (Bragin et al., 1999, 2000, 2004; Traub et al., 2002; Grenier et al., 2003; Dzhala and Staley, 2004; Jacobs et al., 2008; Zijlmans et al., 2009; Brázdil et al., 2010; Haegelen et al., 2013; Wang et al., 2013; Miao et al., 2014). This activity was initially detected in human and animal epileptic tissue and in animals that develop spontaneous seizures (Bragin et al., 1999, 2000, 2004). In the brain, high-frequency oscillations reflect coherent discharges of neurons in response to kainic acid activation of kainate receptors and are generated as a result of sequential and recurrent propagation of action potentials throughout the principal cell population. Using this paradigm, we found that injection of kainic acid (KA, 5 mg/kg, s.c) 15 min after insulin injection (1IU, s.c) led to significant increase in the firing rate, amplitude of extracellular field potentials and PSDs of high frequency brain waves in control mice only (slow and fast ripples: 125–250 and 250–500 Hz, respectively). Pre-injection of insulin prevented the KA-induced increase in ripples activity in the PH-Tau-Tg mice. Increased rates and PSDs of slow and fast ripple serve as a biomarker of epileptogenicity. By all measures used, we found that insulin significantly reduced neuronal excitability and seizure susceptibility in PH-Tau-Tg mice compared to controls (Figures 1–4). Indicating a strong effect of neuronal excitability. To further investigate the alterations in insulin signal transduction pathway in PH-Tau-Tg mice, we used an intraperitoneal glucose tolerance test. We found that PH-Tau-Tg mice have fasting hyperglycemia compared to controls (Figure 6). Hyperglycemia, hyperinsulinemia, and insulin resistance are hallmarks of T2D and has been shown to increase the risk for AD (Sims-Robinson et al., 2010). In this study, we found a significant increase p(Ser)-IRS1 in PH-Tau-Tg mice compared to controls (Figure 5), indicating alteration in IR signal transduction and some levels insulin resistance. Concomitant with the increase in p(Ser)-IRS1 in PH-Tau-Tg cortices, we also found a moderate increase in the expression levels of Glut 4 indicating alterations of glucose transport and utilization in PH-Tau-Tg mice. The expression levels of Gluts 4 have been shown to be dependent on the activation levels of IR. Interestingly, higher level of phosphorylated insulin receptor (p-Ser-IRS1) has been shown to be a consistent change in insulin signaling and a marker of insulin resistance in AD brains (Steen et al., 2005; Moloney et al., 2010; Bomfim et al., 2012; Talbot et al., 2012; Yarchoan et al., 2014). p(Ser)-IRS1 is a substrate for p-JNK, which also has also been found in elevated levels in AD brains (Bomfim et al., 2012; Talbot et al., 2012), suggesting some level of insulin resistance in AD. It has been reported that tau can regulate insulin signaling (Marciniak et al., 2017), and the lack of tau could be the cause of the insulin resistance observed in our system. Despite we cannot completely rule out this possibility, it seems unlikely since our mouse model has the same level of normal tau as the non-transgenic animals and the amount of PH-Tau expression is not more than 14% of that of the endogenous tau. Whether the insulin resistance observed in our mouse model is due to a lack of tau, this will reinforce the idea that PH-Tau has a gain of toxic function by binding to normal tau as we have previously described (Alonso et al., 1996). These findings further validate our mouse model for the study of the correlative relationship between AD and T2D. Consistent with this, a longitudinal study found that fasting hyperinsulinemia, even without T2D, doubled the risk of developing AD (Luchsinger et al., 2004). A cross-sectional study found that in AD patients without an APOE4 allele, hyperinsulinemia was also associated with an increased risk of AD (Kuusisto et al., 1997) and higher insulin was associated with amyloid deposition even before symptom onset (Willette et al., 2015) as shown by amyloid imaging on positron emission tomography scans. Furthermore, analyzes of the relationship between insulin resistance and AD biomarkers during the asymptomatic, preclinical stage in at-risk populations revealed that insulin resistance was associated with higher CSF tau, p-tau (Willette et al., 2015) and Aβ42 (Hoscheidt et al., 2016). Taken together, these studies suggest that alterations in insulin signal transduction pathway could play a causative role in AD. From the literature, an insulin imbalance could trigger tau hyperphosphorylation and then tau-induced neurodegeneration. The results presented here depict a different scenario, where the appearance of pathological tau in a neuron is enough to induce insulin resistance in the brain, that in turn could increase the levels of abnormal tau. It becomes evident that is vital to learn about the function of tau beyond a microtubule associated protein to attempt to design new therapeutics approaches to fight Alzheimer’s disease.

## Data Availability Statement

The original contributions presented in the study are included in the article/Supplementary Material, further inquiries can be directed to the corresponding author/s.

## Ethics Statement

The animal study was reviewed and approved by Institutional Animal Care and Use Committee of the College of Staten Island/CUNY.

## Author Contributions

AA generated and kept the transgenic animals. AA and AE obtained the electrophysiological recordings, immunocytochemistry and glucose levels and prepared the manuscript. AE analyzed the electrophysiological recordings. All authors contributed to the article and approved the submitted version.

## Conflict of Interest

The authors declare that the research was conducted in the absence of any commercial or financial relationships that could be construed as a potential conflict of interest.

## Publisher’s Note

All claims expressed in this article are solely those of the authors and do not necessarily represent those of their affiliated organizations, or those of the publisher, the editors and the reviewers. Any product that may be evaluated in this article, or claim that may be made by its manufacturer, is not guaranteed or endorsed by the publisher.
