# Supplementary material for: Pathological Human Tau Induces Alterations in the Brain Insulin Signaling Cascade
Source: Front Neurosci. 2022 Feb 21;16:805046. doi: 10.3389/fnins.2022.805046 (PMC8899662; doi:10.3389/fnins.2022.805046)
Supplement: Supplementary file 1 [file Data_Sheet_1.PDF]

We have demonstrated that a Pathological human tau form {PH-Tau, R406Wtau pseudophosphorylated at Ser 199, Thr212, Thr231 and Ser262) mimics AD abnormal tau (Alonso et al., 2010), impairs learning and memory in Drosophila (Beharry et al., 2013) and in a transgenic mouse model in which it induces also neuronal death and astrocytes activation (Di et al., 2016). To test the effect of tau hyperphosphorylation on neurodegeneration and cognition we generated a new mouse model in which expression of PH-tau is controlled by the Tet-Off system (SupFig. 1; Di et al. 2016). The Tet-Off system is regulated by the addition (suppressed) or removal (induced) of doxycycline (Dox) to the food and/or water of these animals. By controlling the expression, we can mimic the sporadic form of AD in which tau hyperphosphorylation may be triggered by environmental factors, stress, traumatic brain injury, or another unknown cause. We found that double transgenic mice in which PH-tau is suppressed still expresses baseline levels of PH-tau (~4% of total tau protein). At this low level, PH-tau expression triggers early cognitive deficits in comparison with non-transgenic or single transgenic animals (SupFig. 1H), which may be caused by loss of synapses in the hippocampus (SupFig. 1I). Supporting this, a decrease in the number of synapses was detected by EM in the CA1 region, and the levels of synaptic marker proteins, such as synaptophysin and PSD95 (SupFig. 1J), are significantly reduced in hippocampus. In this condition, we detected localization of PH-tau in the nucleus of neurons (SupFig. 1E and G). Interestingly, this cognitive deficit appears to be more significant than in the mice in which expression of PH-tau is induced (~14% of total tau, SupFig. 1H). To our knowledge, this is the first model where barely detectable levels of abnormal tau can cause dramatic effects.

Upon induction, PH-tau expression correlates with a decrease in brain size (SupFig. 1A), induces tau accumulation and localization to the somatodendritic compartment similar to those found in AD brain (SupFig. 1C-D). PH-tau expression is observed in the forebrain of the mice and results in significant neuronal loss (SupFig. 1E) and astrocytosis (SupFig. 1F). Large scale neuronal death (up to 60% in CA3, SupFig. 1F) and atrophy of the forebrain (SupFig. 1B). Despite the neuronal loss in CA3, the number of synapses in CA1 (SupFig 1 I) and the levels of PSD95 and synaptophysin (SupFig. 1J) are increased compared to animals with low tau expression.

In the present report we set up to investigate the insulin signaling pathway in our mouse model of neurodegeneration in adult mice that express PH-Tau but earlier than when we observed the structural changes in the brain, therefore we chose 8 months of age to test the insulin signaling pathway, and we show that low level of expression of pathological human tau is enough to induce brain insulin resistance.

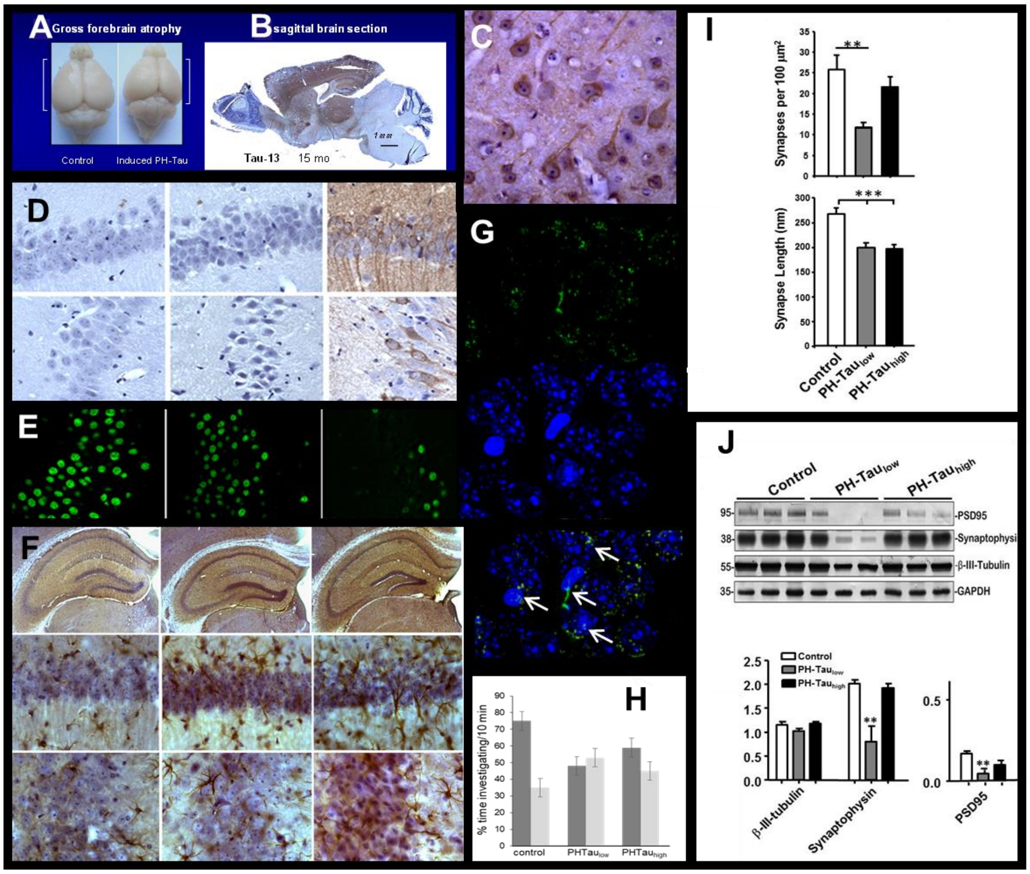

SupFig. 1: Characterization of our novel bigenic mouse model expressing different levels of PH-tau. We generated a new mouse model inducible for a pseudophosphorylated form of tau (Di et al. 2016), expressing PH-tau at 4% of the endogenous tau (PH-tau$_{low}$) and at 14% of the endogenous tau when induced (PH-tau$_{high}$). **A)** Brains of control (left) and PH-tau$_{high}$ (right) mice showing a loss of size when PH-tau is expressed. **B)** Sagittal paraffin section of PH-tau expressing mouse stained with human-tau antibody Tau-13 (brown) and counterstained with hematoxylinne (blue). **C)** From those sections, magnifications of the cortex showing tau mislocalization and accumulation similar to that seen in AD patients. **D)** Magnifications from control (left), PH-tau$_{low}$ (middle) and PH-tau$_{high}$ (right) showing the same staining as in (B). CA1 (upper row) and CA3 (bottom row). Notice that tau staining in PH-tau$_{low}$ is concentrated in the condensed nuclei. **E)** Coronal slices of hippocampus from control (left), PH-tau$_{low}$ (middle) and PH-tau$_{high}$ (right) stained with antibody NeuN recognizing nucleus of neurons, showing CA3 that has a 60% neuronal loss in PH-tau$_{high}$ animals. **F)** Coronal sections stained with GFAP antibody from control (left), PH-tau$_{low}$ (middle) and PH-tau$_{high}$ (right) showing the hippocampus (top row), astrocytes in CA1 (middle row) and CA3 (bottom row) area of bigenic mice brains. Recognized morphological changes, including enlarged size and numerous cytoplasmic processes, are detected. **G)** Immunocytochemistry with antibody Tau-13 (green) and counterstained with DAPI (blue) in hippocampal section of PH-tau$_{low}$ animals showing overlap of tau staining in the nuclei (arrows). **H)** Bigenic mice (12-mth old) were tested for behavior deficits in the Novel Object Recognition task. Significant decreases in spatial memory and memory storage were observed, compare time spent investigating novel object (dark gray bars) with the familiar object (light gray bars). **I)** PH-tau$_{low}$-mice show significant loss of synapses, decreased post-synaptic density and enlarged pre-synaptic portion in electron microscopy staining of the hippocampal sections and quantitation of the number and length of synapses in the CA1 stratum radiatum area**. J)** Representative Western blot of mouse hippocampus homogenate. The protein levels were measured by densitometry and normalized with the levels of GAPDH, showing a marked decrease in the synaptic proteins studied particularly in PH-tau$_{low}$ animals (*P<0.05, **P<0.01, ***P<0.001
